# Validation of eDNA Surveillance Sensitivity for Detection of Asian Carps in Controlled and Field Experiments

**DOI:** 10.1371/journal.pone.0058316

**Published:** 2013-03-05

**Authors:** Andrew R. Mahon, Christopher L. Jerde, Matthew Galaska, Jennifer L. Bergner, W. Lindsay Chadderton, David M. Lodge, Margaret E. Hunter, Leo G. Nico

**Affiliations:** 1 Department of Biology, Institute for Great Lakes Research, Central Michigan University, Mount Pleasant, Michigan, United States of America; 2 Environmental Change Initiative and Department of Biological Sciences, University of Notre Dame, Notre Dame, Indiana, United States of America; 3 Great Lakes Project, The Nature Conservancy, Notre Dame Environmental Change Initiative, Indiana, United States of America; 4 Southeast Ecological Science Center, U.S. Geological Survey, Gainesville, Florida, United States of America; Auburn University, United States of America

## Abstract

In many North American rivers, populations of multiple species of non-native cyprinid fishes are present, including black carp (*Mylpharyngodon piceus*), grass carp (*Ctenopharyngodon idella*), bighead carp (*Hypophthalmichthys* nobilis), silver carp (*Hypophthalmichthys molitrix*), common carp (*Cyprinus carpio*), and goldfish (*Carassius auratus*). All six of these species are found in the Mississippi River basin and tracking their invasion has proven difficult, particularly where abundance is low. Knowledge of the location of the invasion front is valuable to natural resource managers because future ecological and economic damages can be most effectively prevented when populations are low. To test the accuracy of environmental DNA (eDNA) as an early indicator of species occurrence and relative abundance, we applied eDNA technology to the six non-native cyprinid species putatively present in a 2.6 river mile stretch of the Chicago (IL, USA) canal system that was subsequently treated with piscicide. The proportion of water samples yielding positive detections increased with relative abundance of the six species, as indicated by the number of carcasses recovered after poisoning. New markers for black carp, grass carp, and a common carp/goldfish are reported and details of the marker testing to ensure specificity are provided.

## Introduction

Molecular tools are being used for detection of rare species in aquatic ecosystems [1], [2] and are providing actionable information to natural resource management agencies [3]. Dissolved DNA and/or fragments of tissue containing DNA, dubbed environmental DNA (eDNA), remain in suspension for extended periods [1], [4], with DNA remaining detectable in the water column for days to weeks [4]. Consequently, water samples from rivers and lakes can be analyzed for presence of species-specific DNA fragments as a non-invasive method of detection [1], [5], [6]. For example, researchers used eDNA surveillance to delineate the invasion front of bighead (*Hypophthalmichthys nobilis*) and silver carp (*H. molitrix*) in the Chicago area waterway linking the Mississippi River and Great Lakes basins (USA), and demonstrated that eDNA had a greater detection sensitivity than traditional netting and electrofishing sampling methods [2].

Asian carps (or Asian river carps) refer to a group of large-bodied (maximum >1 m long adult size) fish species of the family Cyprinidae that are native to large rivers of eastern Asia. In their native habitat, Asian carps migrate upstream to spawn in large rivers [7]. In addition to the bighead and silver carps, the Asian carp group includes the black carp (*Mylpharyngodon piceus*) and grass carp (*Ctenopharyngodon idella*). These four species, along with two other non-indigenous cyprinid fishes, goldfish (*Carassius auratus*) and common carp (*Cyprinus carpio*), have been introduced to many regions of the world [7], [8]. As a result of escapes and releases, wild populations of these species are now present in various river systems outside of their native ranges. In the North American Mississippi River Basin, Asian carps are either already widespread (e.g., common carp, goldfish, and grass carp) or are rapidly expanding their ranges (e.g., silver carp, bighead carp, and black carp) [7], [9]. Although much about the impacts of some species remains unknown, Asian carps are generally considered ecological and economic threats to North American aquatic environments and fisheries [7], [9], [10], and the surveillance of expanding populations is needed to make informed management decisions [11].

### Animal Welfare Statement

Since we did not utilize any vertebrate animals in this experiment directly (we sampled water and did not house or manipulate any species directly), no IACUC or animal welfare protocol was required for this study.

## Materials and Methods

### Study Site and Piscicide Treatment

**Figure 1:**
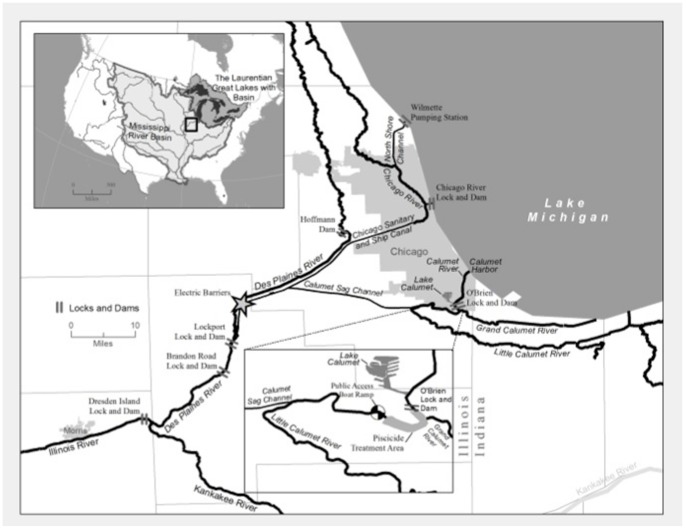
Chicago area waterway system. All field samples reported in this study came from the Little Calumet River at the base of O’Brien Lock. In June of 2010, a bighead carp was recovered from Lake Calumet by the Illinois DNR.

On May 20, 2010, a 2.6 river-mile stretch of the Little Calumet River covering 173 surface acres was treated by a consortium of state and federal agencies with the piscicide rotenone (Fig. 1). This was prompted by repeated detections of bighead and silver carp eDNA from this stretch of the Chicago Area Waterway system, the largest hydrological connection between the Great Lakes and Mississippi River basins (Fig. 1) [2]. Large numbers of fish killed by the rotenone were recovered from the waterway and the identification and counts of the carcasses provided an index of relative abundance of fish species [12]. Using eDNA samples taken in the same stretch of river during the two months prior to poisoning, we investigated whether the proportion of water samples testing positive for eDNA of Asian carps in the CAWS has the same positive association with the relative abundance of fish species recovered after the management action – a trend that has been demonstrated in other field and experimental conditions [1], [13].

### Species-specific PCR Marker Design

We used previously published species-specific Polymerase Chain Reaction (PCR) primers for bighead and silver carp detection [2], and document here the design and testing of novel, species-specific PCR amplification primers for black carp, grass carp, and goldfish/common carp mitochondrial DNA gene fragments. For common carp (*Cyprinus carpio*) and goldfish (*Carassius auratus*), we designed a single primer pair that amplifies both species because of their genetic similarity and the common occurrence of hybridization in wild populations [14].

To develop primers, mitogenomic data were gathered from GenBank (http://ncbi.nlm.nih.gov) for each of the target species along with several native fishes (see below) considered either closely related or that might co-occur with the target species in the Mississippi River Basin. Both black carp and grass carp are the only known species within their respective genera (i.e., monotypic genera), which reduces the likelihood that any native (or other non-native) cyprinids in North America might cross-amplify with the developed markers. For each species, sequence data obtained from GenBank were aligned using BioEdit [15] and a number of sets of putatively species-specific PCR markers were designed *in silico* using the computer software package AlleleID (Premier Biosoft, Inc.) or the Primer-BLAST function in Genbank (http://www.ncbi.nlm.nih.gov/tools/primer-blast/).

Candidate primer sets were screened for positive amplification for their intended target species in the lab using genomic DNA extracts from tissues of the target organisms. Additionally, candidate primer sets were screened on multiple non-target species to ensure specificity of the markers to only intended target species. Targeted PCR amplification reactions were performed on genomic DNA extractions from multiple individuals of target species (black carp, grass carp, goldfish and common carp (n = 3 of each species)). All candidate primer pairs were tested on genomic DNA from non-target species (minimum n = 3 individuals unless noted) including goldfish, common carp, black carp, grass carp, bighead carp, silver carp, fathead minnows (*Pimephales promelas*), largemouth bass (*Micropterus salmoides*; n = 1), gizzard shad (*Dorosoma cepedianum*), and round goby (*Neogobius melanostomus*; n = 2). These closely related and non-closely related non-target species were representative of multiple major taxonomic groups in the region.

Additionally, the functionality of species-specific markers designed to detect black carp and grass carp DNA in water samples was tested with samples collected from ∼3800-liter flow-through aquaculture tanks containing either juvenile grass carp or a mix of grass carp and black carp (Table 1). Consistent with methods described in Jerde et al. [2], 23 two-liter water samples were collected from these tanks with known densities of the target species. Marker specificity was shown for both grass carp and black carp through standard eDNA analyses and detection sensitivities were demonstrated for these two target species. Results (Table 1) show the species-specific molecular markers are functional for eDNA screening, successfully amplify black carp or grass carp DNA irrespective of ploidy (PCR markers designed here do not distinguish between diploid and triploid individuals).

**Table 1:** Screening of the species-specific primers for amplification of captive black carp and grass carp environmental DNA in large, flow-through tanks.

Screening	Contents	Number of water samples	Results	
			grass carp	black carp
Tank 1	∼1000 grass carp (15–20 cm), 10 black carp(30–35 cm)	14	+; 14/14	+; 14/14
Tank 2	∼1000 grass carp (15–20 cm)	4	+; 4/4	−; 0/4
Tank 3	∼1000 grass carp (15–20 cm)	3	+; 3/3	−; 0/3
Tank 4	∼500 grass carp (15–20 cm)	2	+; 2/2	−; 0/2

Results are listed for each sample (number positive water samples out of total water samples). Each test (PCR screening) was replicated 8 times on each water sample; the result was counted as positive for the water sample if at least one of the 8 replicate PCR tests was positive.

Screening of all candidate primer sets resulted in three sets of species-specific PCR primers, one set for black carp, one set for grass carp, and one set for common carp and goldfish combined (Table 2). All primers amplified their target species, and none showed any non-target species amplification.

**Table 2:** Species-specific PCR primer sequences designed to amplify short fragments of mitochondrial DNA of black carp and grass carp, respectively, for this investigation.

Species	Primer name	Primer sequence	Fragment length
*Mylopharyngodon piceus* (black carp)	BLC-COII-F	5'-AAACTTACCAACAAATAC-3'	170bp COII
	BLC-COII-R	5'-TATCATTGATGTCCTATG-3'	
*Ctenopharyngodon idella* (grass carp)	GRC-ND2-F	5'-AATCAATACCTTAGCAATCATTCCA-3'	157bp ND2
	GRC-ND2-R	5'-TATTTATATCTCACTCTCCTGTAAT-3'	
*Carassius auratus* (goldfish)*/Cyprinus carpio*(common carp)	GFCC-COI-F	5'-AGCCCACGCAGGAGCATCAG-3'	171bp COI
	GFGC-COI-R	5'-ACGGCGGTTACAAGCACGGA-3'	

Additionally, the universal amplification marker for common carp and goldfish are included in this table.

All PCR amplification reactions for marker testing and subsequent screening used in this investigation consisted of 0.75U *Taq* Polymerase and 10X PCR buffer (5 Prime), 2.5 mM Mg(OAc)_2_, 10 nmol each of dNTP, DNA template, species-specific primers (0.2 mM final concentration each; Table 2), and deionized water to a total volume of 25 µL per reaction. The PCR thermal program consisted of an initial denaturation at 94°C for 2 minutes followed by 30 cycles of 94°C for 30 seconds, 50°C (52°C for the goldfish/common carp amplifications) for 30 seconds, 72°C for 45 seconds and a final extension of 72°C for 3 minutes. All reactions were screened on agarose gels stained with ethidium bromide, visualized, and photo-documented. For all field sample screenings, each test (PCR screening) was replicated 8 times on each water sample; the result was counted as positive for the water sample if at least one of the 8 replicate PCR tests was positive. Per our previously published methods [2], duplicate sets of 8 reactions (16 more reactions) were run when a sample was found initially positive (1 of 8 reactions or more positives). A sample must screen positive a second time (upon replication) before it is determined to be a confirmed positive for the target species. Additionally, approximately 5% of samples that screened as positive (duplicate times) were validated by sequencing. Every time sequence validation was completed, all successful sequencing reactions matched their intended target species when screened in GenBank.

### Field Application of eDNA Technology for Non-native Carp

We applied the molecular markers designed for grass carp, black carp, and common carp/goldfish to screen water samples previously collected, processed, and analyzed for presence of bighead and silver carp eDNA [2]. As reported in Jerde et al. [2], field samples (2L water samples) were collected from surface waters of the Little Calumet River, covering approximately 2.6 river miles south and west (downstream) of the Thomas J. O’Brien Lock (41° 39′ 08.1″N, 87° 33′ 59.5″W), the portion of river that included the piscicide treatment reach (Fig. 1). Samples were screened for black, grass, and goldfish/common carp from 30 March 2010 and 15 April 2010. Bighead and silver carp eDNA samples were assessed for additional dates: 23 September 2009, 24 November 2009, 8 December 2009, and the day of the piscicide application 20 May 2009. Results were previously reported in Jerde et al. [2]. Differences in the numbers of samples and dates screened for each species are due to the loss of stored samples in a freezer malfunction. Hence, the sample number is zero for grass carp, black carp, and common carp/goldfish for some dates; on 30 March 2010 and 15 April 2012, n = 24 and 7 for common carp/goldfish and n = 33 and 25 for the other carp species, respectively. No samples screened as a part of this effort tested positive for the presence of black carp DNA.

## Results

**Figure 2:**
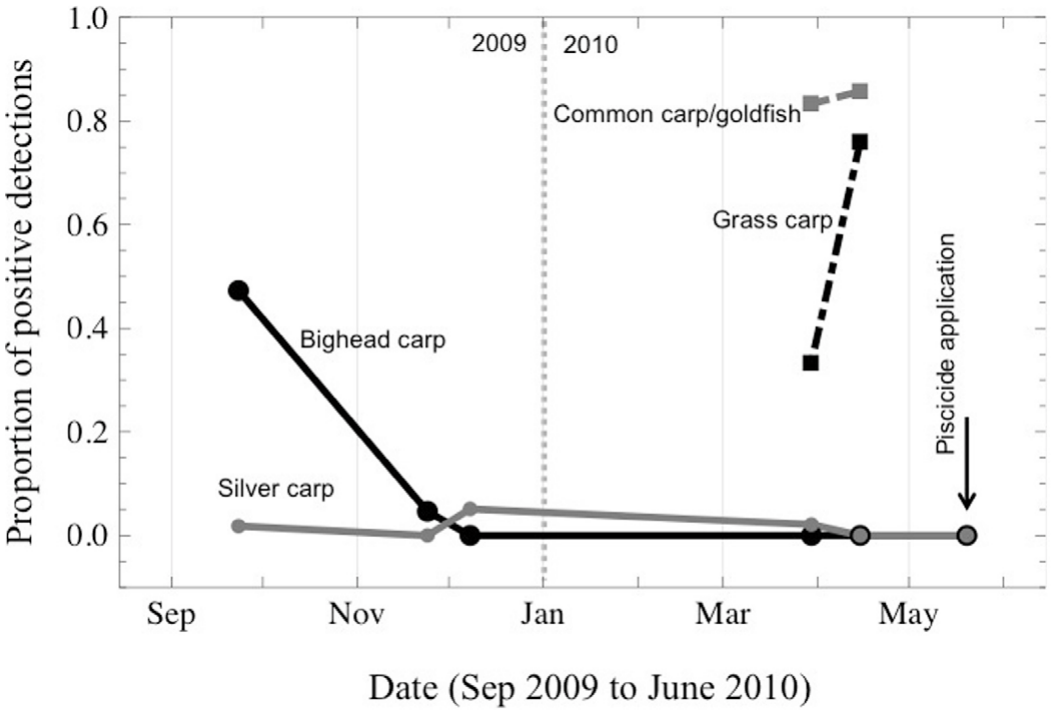
Detection trends of bighead, silver, grass, and goldfish/common carp in the Little Calumet River, in the zone treated with piscicide. Prior to the piscicide treatment, all species except black carp were detected in the treatment reach.

**Figure 3:**
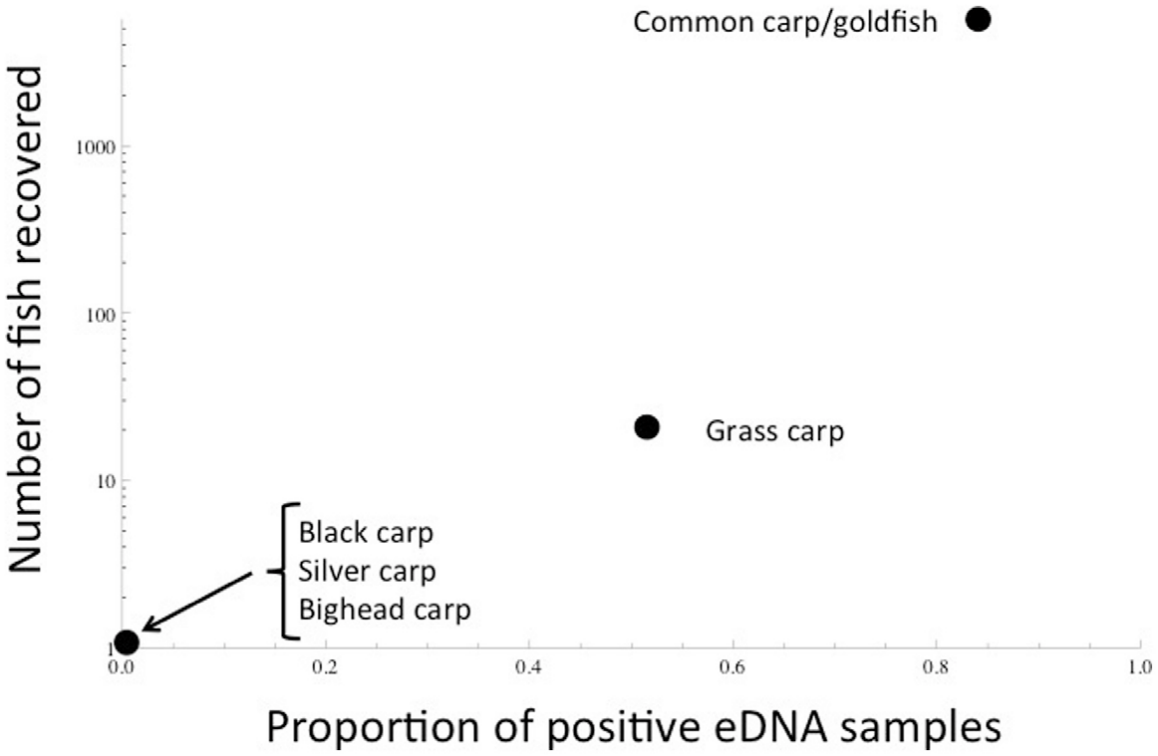
Relationship of detections using environmental DNA and the number of fish recovered in the 2010 rotenone effort.

Prior to the piscicide treatment, both bighead and silver carp were detected in this reach (Fig. 2), including one positive sample for silver carp DNA from March 30 (2010) (1 water sample out of 47 samples), but no silver carp DNA was detected on 15 April 2010 (0/31) or 20 May 2010 (0/46; sampling occurred immediately preceding the rotenone application). Although bighead carp were detected in 2009, no detections were observed in treatment reach in 2010 (Fig. 2). Over half of the tested samples screened positive for grass carp eDNA (51.7%) and 83.9% of the samples tested were positive for goldfish/common carp DNA. After the piscicide treatment, no physical specimens of bighead, silver, or black carp were recovered. However, 21 grass carp (43 estimated) and 5711 (13,081 estimated) common carp, goldfish, and goldfish × common carp hybrids were recovered, the latter constituting approximately 65% of the total biomass recovered [12]. To compare the eDNA results against the numbers of fish recovered, we pooled the eDNA samples across two 2010 sampling dates (30 March, 15 April, 20 May). Although low samples size (5 species) precludes robust statistical analysis of the correlation between proportion of eDNA positives and the number of fish recovered, there appears a positive relationship (Fig. 3). This finding is consistent with recent work showing positive correlation between presence of target organisms and the amount of DNA in the water [1], [15].

## Discussion

### Conservation Impacts

Molecular detection tools, such as eDNA surveillance, can provide an advantage over typical field monitoring and sampling methodologies through increased sensitivity [2], [4], [6]. However, if eDNA techniques are to be broadly applicable for surveillance and monitoring, the molecular markers needed for detection must be species-specific, thoroughly tested, and widely available. In this study, we have added three new genetic markers to the surveillance library, documented their specificity and field application to help promote their use, and increase surveillance efforts for these invasive species. Our field results, along with other recent findings for a variety of aquatic taxa [1], [2], [4]–[6], demonstrate the usefulness of eDNA for detecting the presence of species at low abundance, e.g., grass carp. Additionally, this work supports the growing body of evidence that PCR-based eDNA can provide at least a qualitative index of relative species abundance [1] across similar taxa.

Previous studies have investigated the correlation between aquatic eDNA concentration and organismal biomass [13]. Takahara et al. [15] utilized RT-PCR in controlled monoculture experiments to uncover the relationship between eDNA concentration from one target species and applied their results in a lagoon field study using 21 field samples. Similarly Thomsen et al. [1] reported a correlation between abundance of *Pelobates fuscus* and *Triturus cristatus* in natural ponds. Here we have evaluated the recovery of bighead, silver, black, grass, and goldfish/common individuals during and the similar positive relationship to eDNA detections in a waterway of particular conservation management concern [1], [2], [11], [15]. The building body of evidence from other locations and methodologies and a diversity of species is a robust conclusion that eDNA detection (or concentration of eDNA in a sample) is positively correlated to the abundance and/or biomass of the species [1], [2], [15].

Results we report provide additional guidance for application of eDNA in field surveillance programs. Even when a taxon is present at very high abundance–like the common carp/goldfish–less than 100% of water samples were positive. On the other hand, for other species at low abundance, such as grass carp, a relatively high 51% of water samples were positive although as few as 21 grass carp were present. This could result from non-homogenous mixing, heterogeneity in the distribution of the fishes within the water column, or as Goldberg et al. [6] noted, inefficiencies in DNA extraction and amplification processes. These results suggest that the relationship between eDNA detection and abundance is non-linear (Fig. 3), but definitive conclusions are not possible with the phylogenetically restricted and limited number of species tested.

The lack of detections of bighead, silver, and black carp and the absence of any recovered individuals on the day of the piscicide application serves to punctuate two important points. First, the eDNA method has the potential to be a reliable indicator of the absence of live fish, although we remain cautious in interpreting negative results until more tests are available of the sensitivity of the eDNA method for more species under a range of conditions. Second, alternative pathways by which DNA could be moved (other than a live fish, e.g., feces from fish-eating birds, dead fish deposited off barges), were not apparent in this study; otherwise we would have had positive eDNA detections of bighead and silver carp on the day of rotenone treatment when they were absent from the system. Previously bighead and silver carp had been detected in the treatment reach (Figure 2), and while it is possible that live bighead and silver carps were still present in the area at low abundance, it seems more plausible that previously detected fish had moved from that stretch of river. The large numbers of common carp/goldfish and grass carp recovered in this and a previous treatment in Lockport pool in 2009, suggests the Chicago waterway system provides suitable habitat for these species, especially because previous tracking studies indicate high site fidelity for these species [16]. Other Asian carps are known to move long distances [9] and there is evidence the Chicago waterway system may not be attractive to bighead and silver carp [11]. Any silver or bighead carps dispersing from downstream might be expected, therefore, to pass through the waterway system but not remain resident. Thus, it is reasonable to initiate a comprehensive screening program for bighead and silver carp throughout the Great Lakes, targeting those nearshore habitats and tributaries most likely to foster spawning or resident populations [17].

Although genetic markers presented here were designed for eDNA surveillance of waterways, similar markers could equally be used to identify cryptic species or to verify specific species identifications when morphological characteristics have been compromised (e.g., via decomposition, larvae, eggs, filleting, etc.). In addition, water samples from a variety of sources in addition to natural lentic and lotic habitats could be analyzed for eDNA to determine if target species are indeed present, including reservoirs, farm ponds, ships’ ballast tanks, bait tanks, aquarium imports, and tanks on trucks used to transport live fish.

For example, the US Lacey Act list of injurious wildlife prohibits the importation and interstate transport of live black carp, bighead carp, and silver carp. In addition, most states regulate or restrict use of any existing stocks of black carp and grass carp, including (diploids and triploids) within their borders [8], [18]. Nevertheless, shipments of live fish are occasionally contaminated with prohibited fish species [19]. Verifying the identity of each and every fish can be prohibitively expensive and time consuming because black carp superficially resemble grass carp, the two species have occasionally been misidentified. Indeed, black carp reportedly first arrived in the United States in 1973 as a “contaminant” in one or more transcontinental shipments of other live Asian carp, perhaps grass carp, sent to an aquaculture facility in Arkansas [19]. The results of this study, and the potential for similar efforts on many other species, can facilitate more effective and less expensive surveillance and management of potentially harmful aquatic species.

Finally, environmental DNA surveillance technology is rapidly evolving with refinements of methodology and advances in experimentation [20]. Studies that calibrate species abundances to proportions of detections, or amount of target DNA through qPCR, have the potential to create a powerful tool for resource managers [20]. Additionally, the application of high throughput next-generation sequencing could revolutionize the estimation of species richness and biodiversity [1].
